# Natural History of Untreated Prostate Specific Antigen Radiorecurrent Prostate Cancer in Men with Favorable Prognostic Indicators

**DOI:** 10.1155/2014/912943

**Published:** 2014-02-20

**Authors:** Neil E. Martin, Ming-Hui Chen, Clair J. Beard, Paul L. Nguyen, Marian J. Loffredo, Andrew A. Renshaw, Philip W. Kantoff, Anthony V. D'Amico

**Affiliations:** ^1^Department of Radiation Oncology, Dana-Farber Cancer Institute and Brigham and Women's Hospital, Harvard Medical School, 75 Francis Street, ASB-I L2 Boston, MA 02115, USA; ^2^Department of Statistics, University of Connecticut, 215 Glenbrook Road, Storrs, CT 06269, USA; ^3^Department of Pathology, Baptist Hospital of Miami, 8900 N. Kendall Drive, Miami, FL 33176, USA; ^4^Department of Medical Oncology, Dana-Farber Cancer Institute and Brigham and Women's Hospital, Harvard Medical School, 450 Brookline Avenue, Boston, MA 02215, USA

## Abstract

*Background and Purpose*. Life expectancy data could identify men with favorable post-radiation prostate-specific antigen (PSA) failure kinetics unlikely to require androgen deprivation therapy (ADT). *Materials and Methods*. Of 206 men with unfavorable-risk prostate cancer in a randomized trial of radiation versus radiation and ADT, 53 experienced a PSA failure and were followed without salvage ADT. Comorbidity, age and established prognostic factors were assessed for relationship to death using Cox regression analyses. *Results*. The median age at failure, interval to PSA failure, and PSA doubling time were 76.6 years (interquartile range [IQR]: 71.8–79.3), 49.1 months (IQR: 37.7–87.4), and 25 months (IQR: 13.1–42.8), respectively. After a median follow up of 4.0 years following PSA failure, 45% of men had died, none from prostate cancer and no one had developed metastases. Both increasing age at PSA failure (HR: 1.14; 95% CI: 1.03–1.25; *P* = 0.008) and the presence of moderate to severe comorbidity (HR: 12.5; 95% CI: 3.81–41.0; *P* < 0.001) were significantly associated with an increased risk of death. *Conclusions*. Men over the age of 76 with significant comorbidity and a PSA doubling time >2 years following post-radiation PSA failure appear to be good candidates for observation without ADT intervention.

## 1. Introduction

Based on the prostate specific antigen (PSA) level, biopsy Gleason score, and American Joint Commission on Cancer tumor (T) category, approximately 10%–50% of men will have evidence of disease recurrence at 10 years following external beam radiation therapy with or without concurrent androgen deprivation therapy (ADT) for prostate cancer [1, 2]. In select cases, where men experience both long intervals to PSA recurrence and slow PSA doubling times, a PSA rise reflects a local-only failure and salvage local therapy is an option [3]. For most men with a PSA failure, however, systemic therapy in the form of ADT is considered to delay progression to symptomatic metastatic disease. The timing of initiating ADT following postradiation PSA failure, however, remains an unanswered question. In light of the protracted natural history following PSA failure before the development of clinical symptoms from metastasis [4], as well as the significant morbidity associated with long-course ADT in an aging population with competing risks of death, there is a need to identify patient subsets who may be followed expectantly with serial PSA's and bone scan monitoring without ADT. We hypothesized that using a validated comorbidity metric such as the Adult Comorbidity Evaluation (ACE) 27 Comorbidity index, age, and prognostic factors in the PSA failure state, one may be able to identify men who can avoid ADT and live their life with a high likelihood of no progression to metastases.

Factors associated with improved outcomes following PSA failure after local therapy include prolonged time to failure [5], slower PSA doubling time [6], and lower Gleason score [7]. While a randomized trial (NCT00439751) investigating the timing of ADT initiation following PSA failure after radiation is underway, we currently lack established patient characteristics predicting men who can safely avoid ADT.

We retrospectively studied a cohort of men with unfavorable-risk localized prostate cancer enrolled in a prospective randomized trial of radiation alone or radiation with 6 months of combined ADT where patient comorbidity at baseline using the ACE-27 comorbidity metric was available [8]. We evaluated whether clinical features associated with the risk of death could identify men appropriate for observation without salvage ADT following postradiation PSA recurrence who would not suffer clinical progression to metastases prior to death from a nonprostate cancer cause.

## 2. Materials and Methods

### 2.1. Initial Treatment, Followup and Description of the Study Cohort

The cohort is comprised of men enrolled in a randomized trial of radiation or radiation with 6 months of combined ADT [8]. At enrollment, all men had localized intermediate or high-risk disease (PSA > 10 ng/mL or Gleason ≥ 7 and 2002 AJCC clinical T category T1b to T2b) and workup included central pathology review (AAR), PSA value, bone scan, and computerized tomographic or magnetic resonance imaging of the pelvis. The radiation was delivered using a three-dimensional, two-phase approach to the prostate and seminal vesicles to a total of 70.35 Gy over 36 fractions. Combined androgen blockade consisted of two injections of an LHRH agonist (leuprolide acetate 22.5 mg every 3 months or goserelin 10.8 mg every 3 months) and a nonsteroidal antiandrogen (flutamide 250 mg every 8 hours or bicalutamide 50 mg daily, discontinued on day 85 after the second administration of the LHRH agonist). Baseline comorbidity at the time of study enrollment was characterized using the ACE-27 instrument [9]. Prior to PSA failure, men were seen in followup with PSA every 3 months for 2 years, every 6 months until 5 years, and annually thereafter.

Of the initial 206 enrolled in the randomized study, 108 (52%) had evidence of a biochemical failure defined as a 2 ng/mL elevation above the lowest PSA value achieved. Per protocol recommendation, 53 of those men who had a PSA that rose to >10 ng/mL were started on salvage ADT for life. Two men underwent salvage brachytherapy for a local only recurrence. The remaining 53 participants with PSA recurrence did not receive salvage ADT either because their PSA remained <10 ng/mL (*n* = 41) or because of significant comorbid illness (*n* = 12) and constitute the study cohort. At the time of PSA failure, all men had restaging with bone scan. Thereafter, followup and restaging were at the discretion of the treating physician and generally PSAs were obtained annually and bone scans were obtained for symptoms. Cause of death was determined by the treating oncologist who followed the patient from study entry until death. This retrospective analysis of the prospectively collected study data was approved by the institute institutional review board.

### 2.2. Statistical Methods

#### 2.2.1. Patient Characteristics Stratified by Survival Status

We used descriptive statistics including median values and interquartile range (IQR) to characterize the study cohort stratified by survival status. Comparisons of categorical covariates were made using a Fisher exact test. Continuous covariates were compared using a Wilcoxon rank sum test [10]. The interval to PSA failure was calculated from the date of randomization and the PSA doubling time was calculated using at least two PSA values >0.2 ng/mL assuming first-order kinetics.

#### 2.2.2. Risk of All-Cause Mortality

Univariable and multivariable Cox regression analyses [11] were performed to identify factors associated with the risk of death following PSA failure. We included known patient, treatment, and previously identified prognostic markers in the model including initial study treatment arm (radiation alone versus radiation plus ADT), PSA doubling time (continuous), biopsy Gleason score (≤7 versus ≥8), clinical T category (T1 versus T2), age at PSA failure (continuous), ACE-27 comorbidity (none or minimal versus moderate or severe) [9], and interval to PSA failure. Both the PSA doubling time and time to PSA failure were log-transformed and treated as continuous measures. Baseline groups for the categorical variables included radiation alone treatment arm, biopsy Gleason 7 or less, T1, and no or minimal comorbidity. Unadjusted and adjusted hazard ratios (HR) and associated 95% confidence interval (CI) were calculated for each covariate.

#### 2.2.3. Estimates of All-Cause Mortality

One minus Kaplan-Meier estimates [12] of overall survival were calculated to estimate all-cause mortality following PSA failure and were graphically displayed stratified into three groups using two factors: the median age at PSA failure and the presence of moderate to severe comorbidity versus no or minimal comorbidity using the ACE-27 metric. Comparisons of the estimates of all-cause mortality between groups were made by log-rank test.

All *P* values are two-sided and Bonferroni corrections [13] were made for multiple comparisons. All analyses were performed using SAS software (version 9.3; SAS Institute, Cary, NC).

## 3. Results

### 3.1. Patient Characteristics Stratified by Survival Status

For the entire study cohort, the median age at PSA failure, interval to PSA failure and PSA doubling time were 76.6 years (IQR: 71.8–79.3), 49.1 months (IQR: 37.7–87.4), and 25 months (IQR: 13.1–42.8), respectively. Over a median follow up of 4.0 years (IQR: 2.0–12.5) following PSA failure, we observed 24 (45%) deaths, all of causes other than prostate cancer and no man had evidence of metastatic disease. The median last followup PSA was 3.6 ng/mL (IQR: 1.8–8.3).

As shown in Table 1, men dead at last followup had a median age of 77.3 years at PSA failure compared to 75.7 years for those alive (*P* = 0.1). The men who had died were more likely to have moderate to severe comorbidity (41% versus 7%; *P* = 0.004) and more commonly had biopsy Gleason ≥7 (92% versus 62%; *P* = 0.02). We did not find that other prostate cancer specific prognostic factors were differential between those who died and those alive at last followup including PSA doubling time (20.3 versus 32.9 months; *P* = 0.55), interval to PSA failure (47.2 versus 57.4 months; *P* = 0.44), and most recent PSA level (2.4 versus 4.7 ng/mL; *P* = 0.16).

### 3.2. Risk of All-Cause Mortality

On univariate analysis (Table 2), factors significantly associated with death in the cohort of men with PSA recurrence but no salvage ADT use were age at PSA failure (HR 1.12; 95% CI: 1.02–1.22; *P* = 0.01) and the presence of moderate to severe comorbidity (HR 5.32; 95% CI: 2.31–12.3; *P* < 0.001). Similarly, on multivariate analysis (Table 2), only age at PSA failure (AHR: 1.14; 95% CI: 1.03–1.25; *P* = 0.008) and the presence of moderate to severe comorbidity (AHR: 12.5; 95% CI: 3.81–41.0; *P* < 0.001) were significantly associated with the risk of death.

### 3.3. Estimates of All-Cause Mortality

**Figure 1:**
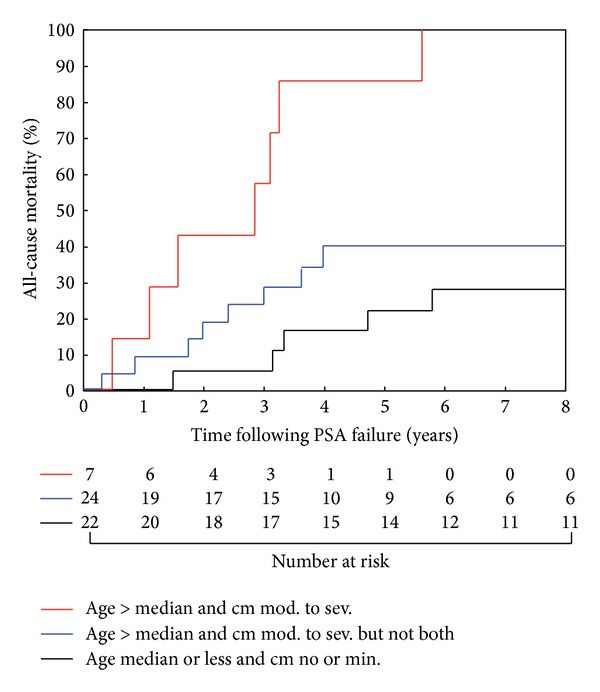
One minus Kaplan Meier estimates of all-cause mortality following PSA failure for subgroups based on the median age at failure (76.6 years) and presence or absence of ACE-27 defined moderate to severe comorbidity (CM). Men older than the median at the time of PSA failure and who had moderate to severe comorbidity were significantly more likely to die compared to men with only one of those features (*P* = 0.003) or men who had neither adverse feature (*P* < 0.001). No significant difference was identified between men with one or neither risk factor (*P* = 0.14). Significance is defined as *P* < 0.0167 per Bonferroni correction.

For the purposes of illustration, we subdivided the cohort into three groups based on the median age at failure and the presence of moderate to severe comorbidity and plotted the cumulative incidence of death (Figure 1). Men older than 76.6 years at the time of PSA failure and who had moderate to severe comorbidity were significantly more likely to die compared to men with only one of those features (*P* = 0.003) or men who had neither adverse feature (*P* < 0.001). Specifically, 4 years following PSA failure, 85.7% (95% CI: 53.5%–99.3%) of men above the median age and with moderate or severe comorbidity were dead compared to 16.4% (95% CI: 5.6%–42.7%) of men younger than the median with no minimal comorbidities. For those men with either age greater than the median or moderate or severe comorbidities, but not both factors, the 4-year mortality was 39.2% (95% CI: 22.1%–64.6%). We could identify no difference between those without either adverse feature and those with only one (*P* = 0.14).

## 4. Discussion

In this study, we identify a subset of men with PSA only recurrences following radiation with or without 6 months of ADT for unfavorable-risk and localized prostate cancer who are unlikely to progress to metastatic disease during their remaining life expectancy despite withholding salvage ADT. Specifically, we show that men with both a long interval to PSA failure (median 49 months) and long PSA doubling time (median 25 months) who are advanced in age (median 76.6 years) with moderate to severe comorbidity appear unlikely to progress to symptomatic distant metastatic disease or die of prostate cancer. Only 14% of this population was estimated to remain alive 4 years following PSA failure despite withholding salvage ADT and all deaths were nonprostate cancer related.

In a healthy cohort selected for radical prostatectomy the median time from PSA failure to metastasis is 8 years and to death from prostate cancer is more than 10 years [4]. Therefore, a better understanding of who, particularly those with limited life expectancy due to comorbidity, may safely avoid treatment with salvage ADT is needed. Randomized trials investigating the role of early versus delayed initiation of ADT in men found at prostatectomy to have lymph node involvement [14] and those with advanced prostate cancer [15] showed a survival advantage to the early initiation of ADT. How these and other results from men with advanced disease translate to the population with a rising following radiation is unclear. Lacking results from a randomized Canadian trial of early versus delayed ADT in men with PSA failure following radiation (NCT00439751), decisions are typically made today in the context of comorbidity and adverse prostate cancer prognostic factors such as PSA doubling time and time to PSA failure.

Using these existing prognostic factors to guide treatment, other groups have reported outcomes similar to ours. Faria and colleagues reported on a cohort of 285 men who underwent external beam radiation with or without ADT and experienced a biochemical failure [16]. Using an approach similar to the one we report here of avoiding salvage ADT in men with long intervals to failure (median 30 months) and slow doubling times (median 26 months), they show that among 113 men with these characteristics, none had developed metastatic disease or died of prostate cancer with nearly 4 years median followup. Klayton and colleagues report on a cohort of 432 men with a biochemical failure following external beam radiation with more than 3 years of followup from failure [17]. They found that salvage ADT was associated with improved prostate cancer mortality only in those men with a PSA doubling time <6 months. A prior publication from this cohort had reported the development of distant metastatic disease in only 8% of 89 men with PSA doubling times >12 months who were not treated with ADT after biochemical failure [18]. These studies did not investigate the role of comorbidity.

While this study is strengthened by the use of prospectively collected data on men who were enrolled in a randomized trial, it has several potential limitations. First, the decision to start or withhold ADT following PSA failure was based on a PSA level of 10 ng/mL without regards to the PSA doubling time since the prognostic value of PSA doubling time was not appreciated at the time the study was designed in 1994. We attempted to adjust for this issue by including PSA doubling time in the multivariable model. Second, with 53 men, the study is not large enough to draw definitive conclusions about all men in whom salvage ADT can be withheld; in older and less healthy men, the data appear robust enough to make this recommendation. While the follow-up time after PSA failure was relatively long at a median of 4 years, it remains to be seen how durable the observed nonprogression to symptomatic disease will be, especially in younger and healthy patients. Third, we used the median age in our model based on statistical standards but at age 76.6 years, the median remaining life expectancy (RLE) for men in average health is approximately 10 years based on social security actuarial life tables lending credence to making a recommendation to withhold ADT in this group of men with favorable prognostic factors and moderate to severe comorbidity where RLE would be expected to be less than 10 years. Finally, the comorbidities were assessed at enrollment in the study and therefore may not perfectly match those present at the time of PSA failure; however, the fact that the ACE-27 score was significantly associated with the risk of all-cause mortality on multivariate analysis is reassuring. Whether the interaction between comorbidity and ADT toxicity can be modified by lifestyle changes is yet to be tested but has the potential to change our observations.

In summary, our data suggest that older, less healthy men with both long intervals to PSA failure and PSA doubling times can safely be spared the morbidity of life-long ADT following postradiation PSA failure. Given the proposed interaction between comorbidity and ADT use [19], withholding ADT in these men may actually prolong their life span. Ultimately, it will take the results of randomized trials where comorbidity is stratified for to definitively answer the question of when and in whom ADT is beneficial following PSA failure.

## Figures and Tables

**Table 1:** Comparison of the distribution of the clinical characteristics at the time of initial treatment and at PSA failure for the 53 men in the study cohort stratified by survival status at time of last followup.

Characteristic	Alive, *n* = 29	Dead, *n* = 24	*P*
Age at PSA failure—median (IQR), yrs	75.7	77.3	0.10
PSA	(69.4–78.6)	(75.0–80.8)	
Doubling time—median (IQR), mo	32.9 (15.0–43.0)	20.3 (10.5–42.6)	0.55
Interval to failure—median (IQR), mo	57.4 (35.0–99.0)	47.2 (38.3–57.9)	0.44
Last PSA level—median (IQR), ng/mL	4.7 (2.9–10.0)	2.4 (1.7–6.9)	0.16
Primary treatment—*n* (%)			
Radiation	19 (66)	16 (67)	1.0*
Radiation + ADT	10 (34)	8 (33)	
Gleason—*n* (%)			
≤6	11 (38)	2 (8)	0.02*
7	16 (55)	16 (67)	
8–10	2 (7)	6 (25)	
T category—*n* (%)			
T1	13 (45)	10 (42)	1.0*
T2	16 (55)	14 (58)	
Comorbidity—*n* (%)			
None or minimal	27 (93)	14 (58)	0.004*
Moderate or severe	2 (7)	10 (42)	

PSA: prostate specific antigen; IQR: interquartile range; ADT: androgen deprivation therapy.

*Fisher exact test *P* value.

**Table 2:** Unadjusted and adjusted hazard ratios for all-cause mortality following PSA failure.

Clinical factor	Number of men	Number of events	Univariable HR (95% CI)	*P*	Multivariable AHR (95% CI)	*P*
Treatment arm						
Radiation	35	16	Ref.	—	Ref.	—
Radiation + ADT	18	8	1.01 (0.43–2.37)	0.99	1.69 (0.68–4.19)	0.26
Gleason						
≤7	45	18	Ref.	—	Ref.	—
>7	8	6	2.02 (0.80–5.10)	0.14	0.82 (0.28–2.44)	0.72
Clinical T category						
T1	23	10	Ref.	—	Ref.	—
T2	30	14	0.93 (0.41–2.13)	0.87	0.65 (0.25–1.72)	0.39
ACE-27 comorbidity						
None or minimal	41	14	Ref.	—	Ref.	—
Moderate or severe	12	10	5.32 (2.31–12.3)	<0.001	12.50 (3.81–41.0)	<0.001
Age in years at PSA failure	53	24	1.12 (1.02–1.22)	0.01	1.14 (1.03–1.25)	0.008
Interval to PSA failure in months*	53	24	1.73 (0.75–3.98)	0.20	2.36 (0.89–6.26)	0.09
PSA doubling time in months*	53	24	0.98 (0.60–1.59)	0.92	1.15 (0.63–2.08)	0.66

*Log-transformed.

ADT: androgen deprivation therapy; PSA: prostate specific antigen.
